# Report on Cholera

**Published:** 1873-09-01

**Authors:** T. F. Worrell

**Affiliations:** President Illinois State Medical Society


### REPORT ON CHOLERA.

READ BEFORE THE M'LEAN COUNTY MEDICAL SOCIETY, AUGUST 4TH, 1873, BY T. F. WORRELL, M.D., PRESIDENT ILLINOIS STATE MEDICAL SOCIETY.

*Mr. President, and Gentlemen of the McLean County Medical Society:*

In response to your action appointing me to prepare a paper on the subject of Cholera, I am here to submit some reflections which embody the views I entertain in regard to the *nature* and the pathology of this disease. I have chosen to discuss this department of the subject because of its *intrinsic* importance, as well as because of the fact that medical men are divided in their opinions and conclusions in reference to the *nature* of this great scourge of our race.

It would, perhaps, be interesting to dwell a little upon the *history* of this terrible epidemic, which, it is believed, originated in the fourteenth century, and which was called "*Black Death*," or "Oriental Plague." The *ravages* of this disease are unparalleled in the history of the epidemics which have depopulated the world. Historians tell us that it swept off one-fourth of the inhabitants of the Old World in the brief period of four years. England lost one-half of its population; London lost 100,000 in four months, and other cities in proportion. It is said that China lost 13,000,000 of her inhabitants by this plague in one visitation. India was almost depopulated; and Tartary and Syria, were literally covered with dead bodies; and ships were seen, long after the subsidence of the epidemic, floating about without crews, in the waters of the Mediterranean. No climate is a barrier to its progress. It made its first appearance on our continent in 1832, with fearful mortality; and though its home seems to be on the Ganges, yet it occasionally visits the inhabitants of all parts of our planet, and leaves a broad mark of devastation and death. Truly, it has a wonderful history, with which you are all familiar; and so I will dismiss its further consideration.

Nor will I occupy your time by any statements in regard to the *diagnosis* of this disease. When it is fully developed, it cannot be mistaken for, nor confounded with, any other of the maladies which afflict and decimate the race. Its symptoms are pretty uniformly the same everywhere, in every clime, with every people.

And before I *state* the theory which I have adopted of its pathology and treatment, I think it proper to quote the opinions of some of the distinguished authors who have written upon this subject. Dr. Thayer, of Brooklyn, who had charge of the Cholera hospitals of that city in '49, '54 and '56, says: "The *essential* pathology of the disease consists in a remarkable change in the blood, by which its red corpuscles lose their power of oxygenation, and there is, consequently, a general arrest of the circulation. He says it is highly probable, that calomel acting primarily on the portal circulation through the intestinal mucous membrane, relieves, secondarily, the systemic capillaries, thereby arresting the destruction of the red corpuscles, while it eliminates the choleraic poison by the bowels;" and he refers to the lamented Nuinger in support of these views.

Dr. Flint says: "The first appreciable result in the history of the disease is transudation into the alimentary canal. The characteristic dejections, and the contents of the intestines found after death, consist of water in great abundance, holding in solution a little albumen, and several saline constituents, the most abundant being the chloride of sodium and desquamated epithelium. The blood, of course, loses what is transuded into the alimentary canal. From this loss of water, the blood becomes dense and viscid. The blood globules are relatively in excess, owing to the loss of water. The blood is deficient in saline elements, which have escaped with the transuded water. The fibrin is not diminished. The albumen is relatively greater than in health; but it is actually diminished."

Most of the striking phenomena of the disease are plainly attributable to the blood lesion *resulting* from the loss of the constituents found in the choleraic dejections.

*The water which does not enter into the constitution of the albumen and fibrin, serves to hold the various salts in solution*, and it cannot vary much in quantity, from a certain standard, without great danger to life. The alkaline carbonates have a tendency to preserve the fluidity of the fibrin. The blood, deprived of its fluid constituents, circulates with difficulty, and the changes effected by respiration take place imperfectly. The transudation, which is first in the catenation of appreciable events, involves an underlying, and, at present, inappreciable, pathological condidition. The latter should be understood in order to know fully the pathological character of the disease.

It is probable that the primary essential change is in the blood, holding in solution, saline constituents, which causes transudation into the alimentary canal.

According to Robin, "this pathological change is an isomeric modification of the coagulable constituents of the blood plasma, in consequence of which, they part with the water entering into their composition; hence the transudation by exosmosis, and defective absorption or endosmosis."

Watson says: "The examination of the dead body throws no light upon the nature of this disease; the principal lesion being that the veins are clogged with thick, black, tar-like blood." Having speculated at some length on the courses of the disease, he concludes in this language: "Its visitations are so rapid, widely spread, and multitudinous, that there is no time for its transferrence from house to house, or from person to person. Its inherent rate of locomotion outstrips and precludes the tardier conveyance of the poison by man. Its contagious qualities (granting that they exist) are hidden in its universality, and can seldom be traced, except by accident.

My own creed is, that cholera is contagious, in a limited sense; at the same time, I believe that a great majority of the cases are not attributable to direct contagion, but to the poison diffused in the atmosphere."

Dr. Clymer adds: "During the prevalence of cholera in Philadelphia in 1832, we closely investigated every fact calculated to throw light upon its contagious or non-contagious character. [For this investigation, our position in the Board of Health, as chief of a large hospital, afforded us ample opportunity.] But we were unable to discover the slightest evidence of the disease having been, in any instance, communicated from the sick to the well."

Aitkin, commences his very full and able article on this disease by the following definition, viz.: "A disease essentially of miasmatic origin, developed under certain atmospheric or territorial local conditions, in Europe, Asia and America, and capable of being propagated or diffused to a certain extent over the surface of the earth, through the atmosphere, or in some other way, and also by means of human intercourse between the healthy and the sick."

On the pathology of this disease, volumes have been written during recent years, many of them by those who have witnessed the behavior of this disease in its native home, as well as in other parts of Asia and Europe; and they all agree that, whatever may be the *exciting* cause or causes, the remote cause is a poison capable of propagation and diffusion, in countries whose climate, soil, and geological formations, as also the moral and physical habits of the population, are as different as possible.

Aitkin says (page 576, vol. I.): "One doctrine, now generally accepted, regarding the pathology of cholera, is, that a poison has been absorbed, and infects the blood; that, after a longer or shorter time, it produces a primary disease of the blood; that it undergoes enormous multiplication in the human body, as a result of the morbid process so established; and that changes are induced, consequent upon the alteration of the blood by the poison." In support of this view, he quotes the great English poet, who, describing this disease, says: "Where effect holds such an enmity with blood of man, that, quick as quicksilver, it courses through the natural gates and alleys of the body, and with a sudden vigor it doth posset and curd, like eager droppings into milk, the thin and wholesome blood."

"But," continues Aitkin, "another doctrine is now also making progress—that the blood is only *secondarily affected*, and that *primarily*, a poison makes its way to the intestinal, mucous membrane, and *there* develops itself to the rapid annihilation of the power of life."

If such a doctrine prove true, then this disease must be classified among parasitical poisons, and treated accordingly. "The phenomena resulting from the changes in the blood are the proper and distinctive symptoms of the disease." (Page 576, bottom.) "The follicular structure of the bowel is found to be swollen and flabby, as is the skin corrugated and sodden, both having been drenched and bleached by a very similar process." The serum poured into the bowels differs but little from that liquid which so abundantly exudes from the cutaneous surface.

In regard to the pathology of cholera, there are but two theories (in the books) which, in my humble judgment, are entitled to merit. The first one asserts that cholera *begins* as an actual blood changer, capable of producing all the phenomena which are observed in the behavior of this disease. The second teaches that it *begins* as a bowel disease, producible by direct contagion with the mucous surface, *without* the intervention of the blood.

That it may be introduced into the system through the medium of the mucous membrane of the bowels, we will admit. But that this is the *only* or usual mode of infection, we are forced to deny. I think it would be utterly fallacious to attribute the numerous cases which occur in different portions of the same community, simultaneously, to any cause acting *primarily* on the mucous membrane of the alimentary canal. *Since* that could be effected in no other way except by eating or drinking that to which the poison had been imparted.

On the other hand—as all inspire a common atmosphere, and as the doctrine that many poisons more or less subtle are conveyed into the circulation by respiration, is accepted by all without controversy.—I cannot see why it may not be the medium of communication for this disease. A very strong and (I think) unanswerable argument in support of this theory is, that a large proportion of the cases come on and are developed too suddenly to admit of the probability that it is caused by a poison making its primary impression on the bowels; for it requires no discussion to prove that those tissues through which oxygen is easily conveyed into the blood, and carbonic gas removed from it, will readily transmit minute particles of poison, whether called ozone or protozone.

I conclude that, in the production of cholera, two factors are required, the one, some air-born material, or some dynamic modification of atmospheric elements coming from *without;* the other, some *local* element; and neither being potential for the production of the disease alone.

I will here undertake to define my view of the *modus operandi* of the poison in the development of those phenomena that constitute the disease called cholera. When the poison has entered the circulation, its first effect is so to alter the blood as to cause its *dissolution*, breaking up that chemical affinity, or rather those chemical and catalitic affinities, by whose influence the several constituents of the blood are kept in their normal, proportionate relations. The serum of the blood does not transude through the vessels, *ordinarily*, simply because it is admixed with the other ingredients of that fluid, forming with them a homogeneous liquid; and also because this union of elements is constantly preserved by those vital or chemical or physiological agencies, which may be denominated the *vis vitæ*, if you please. Sometimes the power of the poison thus introduced is so deadly that it causes an exudation of the red globules of the blood, as we see after a bite by a rattlesnake or tarantula. It would seem that, in this case, the denser portions of the blood were rendered as liquid as its serum, or as though its vital proportion were destroyed by the employment of heat.

At the same time that this great change is produced in the blood plasma, another effect of the poison is displayed; that is this, the tonic contractility of the arteries, veins and capillaries, by whose agency, in part, the blood is carried forward in its circuit through the system, and by which, also, exosmosis is prevented, is suddenly paralyzed. This causes so much relaxation of the blood vessels, that they readily yield to the tendency of the more fluid portion of their contents to escape from them.

The blood, thus deprived of that portion of it which contributes so greatly to its easy and rapid circulation, and also of that vital power which serves a constant stimulus to the performance of this great function of the animal economy, becomes clogged in its course; and if this condition is not speedily relieved, it will cease to flow altogether, and life become extinct as a necessary consequence.

Argument and illustration might be multiplied (as I conceive), to prove that the theory (conjecture, if you please to call it so) which I have adopted, and which I have briefly, but I hope clearly, set forth in this paper, is at least as rational as any that has been advanced; that it accounts for the symptoms as well as for the *post mortem* phenomena characteristic of this dread disease, wherever it has prevailed around the globe; and that it is in perfect harmony with that general mode of treatment which is the practice of all kinds and classes of physicians the world over. I refer to the universal employment of opium as the great sheet-anchor in the treatment, and to alcohol, both of which are always administered, whatever else may be given or omitted. Now, *how* do these great agents operate to control or modify the potential influence of the poison of cholera? I answer, they operate in perfect harmony with the theory I have set up of the pathology of the disease; *i. e.*, they arrest, or at least *retard*, that rapid metamorphosis which is going on in the blood plasma, by their SEDATIVE influence upon the action of those vital functions, by which life is not only preserved but by which also the *virus* of this epidemic is rendered destructive. We all know that small, *stimulating* doses of these remedies, however frequently administered, have but little effect to arrest this disease; and as the powers of life give way rapidly under the overwhelming force of this fell destroyer, it would seem that the first and paramount indication of treatment is to *stimulate* all the failing functions of life. But instead of this, we use the most potential *sedatives*, in potential doses; and we succeed in this way, and in no other.

Another thought, and I shall be done. The *duration* of the poison of cholera in the system, according to Dr. Sutton, is from twenty to thirty hours; that is, the cholera process runs its course and expands all its force in about that time; after that it is inert.

*Then*, if we can cripple its energies or retard its progress by means which, though they depress and partially paralyze the vital functions for a brief period, yet husband nature's recuperative resources, we shall accomplish much to diminish the mortality of a disease whose dreadful nature is portrayed in the sacred Scriptures, where it is spoken of as the "pestilence that walketh in darkness, and the destruction that wasteth at noon-day."

